# Does coronavirus pose a challenge to the diagnoses of anxiety and depression? A view from psychiatry

**DOI:** 10.1192/bjb.2020.102

**Published:** 2020-09-03

**Authors:** Lynne M. Drummond

**Affiliations:** South West London and St George's Mental Health NHS Trust, UK

**Keywords:** Anxiety, depression, COVID-19, coronavirus, SARS-CoV-2

## Abstract

Some authors have suggested that the emergence of the novel coronavirus, SARS-CoV-2, and the subsequent pandemic has meant that the constructs of pathological anxiety and depression are meaningless owing to widespread anxiety and depressive symptoms. This paper examines what is required to make a diagnosis of a depressive or anxiety disorder and how this may differ from fleeting symptomatology in response to specific situations or stimuli. All people experience the emotions of both anxiety and depression, but far fewer have a persistent anxiety or depressive syndrome which interferes with their quality of life and functioning. The pandemic and its issues are then discussed, and existing studies examining the reactions of people living through the pandemic are presented. Finally, the paper examines possible ways to cope at times of increased stress and how we can try to protect ourselves from long-term mental health sequelae of chronic stress.

One of the challenges of conducting studies to examine the prevalence and frequency of anxiety and depression is that these terms describe symptoms rather than a diagnosis. Everyone has experienced these as symptoms at some point in their lives. Diagnoses are made on the basis of a recognised cluster of symptoms associated with anxiety and depression. One of the difficulties is that these terms are also used in general parlance to describe short-lived emotional changes which can occur as an emotional reaction to a stimulus or event, rather than a full-blown syndrome that affects the individual's quality of life. Indeed, if we as a species did not experience anxiety responses in threatening situations, we would undoubtedly have become extinct as we walked over cliff edges, faced up to dangerous carnivores, or generally partook in dangerous and risky behaviour. Levels of anxiety and depression also vary between individuals without necessarily being pathological. For example, we will all know some members of our social circle who are thrill-seekers and attracted to danger, whereas others are much more risk averse. Similarly, some people seem to be happy and philosophical at all times, whereas others are more pessimistic and prone to feel low and miserable at the slightest upset. Overall, therefore, population studies are fraught with difficulties in accommodating this area of potential self-reporting bias. For a diagnosis of anxiety disorder or depression to be made, an individual should be experiencing a range of symptoms associated with the anxiety and depression, and the symptoms should be apparent for a period of time (not just hours or minutes but weeks or months) and sufficiently severe that they interfere with the person's ability to function fully in their home, work, social or private leisure settings. The consistency of the emotional symptoms is inherent in diagnostic instruments such as the ICD,^1^ and widely different prevalence figures may be obtained in self-report questionnaires unless the same criteria are applied.

In medical and psychological parlance, stress is a physical, mental or emotional factor that causes bodily or mental tension. Stresses can be external (from the environment, psychological factors or social situations) or internal (e.g. illness). Stress, both recent and in childhood, is known to affect mental well-being. The coping ability of the individual affects whether or not stress results in deterioration of mental well-being.^2^ Different individuals subjected to similar trauma and stress do not all react in the same way; whereas some will experience lasting symptoms, other will seem to have hardly any lasting sequelae.

Population studies examining the prevalence of anxiety and depression have demonstrated that these are among the most common conditions in diverse societies. The World Health Organization (WHO) estimated that in 2015 the prevalence of depression was 4.4% globally; however, this figure varied with gender, with women having a prevalence of 5.1% and men 3.6%. Depression also increased with age in adulthood, peaking in both genders at age 55–74 years, and varied among different countries. The lowest rate of 2.6% was found in men in the Western Pacific region, and the highest rate of 5.9% in women in the African region. These rates of depression were found to have increased by 18.4% since 2005.^3^ For anxiety, the prevalence was estimated to be 3.6%. As with depression, anxiety disorders are more common among females than males (4.6% compared with 2.6% at the global level). Again, anxiety disorders were found to have increased since 2005 by 14.6%. As depression and anxiety are often comorbid disorders, it is not accurate to combine these two figures.^3^

A recent large study in the UK examined adults for a range of physical and mental disorders (BioBank UK). A questionnaire asking about mental disorders found that 21.2% had received a diagnosis of depression at some time in their lives and 14% had been diagnosed with anxiety. The sample who answered the questionnaire were not representative of the whole population, as the participants were aged 45–82 years, with 53% aged 65 or over, 57% were female and 45% had a degree (compared with census data in which 23% had a degree).^4^

## History of SARS-CoV-2

On 31 December 2019, the WHO was informed of an outbreak of pneumonia of unknown cause in Wuhan City, in Hubei Province, China. It was then announced on 12 January 2020 that a novel coronavirus had been identified in samples from infected individuals. This virus was referred to as SARS-CoV-2 and the associated illness as COVID-19. China acted by completely closing down Wuhan and Hubei Province, but this was not before the virus seemed to have travelled widely globally. In the UK, people arriving from Wuhan or those believed to have been in contact with SARS-CoV-2 were quarantined, but transmission within the UK and people affected by the virus who had not been abroad was first documented on 28 February 2020. The WHO declared a pandemic on 11 March. On 15 March, following observations of a larger outbreak in northern Italy, the UK government asked people to work from home if possible, avoid unnecessary travel and avoid contact with others. Anyone with symptoms suggestive of SARS-CoV-2 was asked to self-isolate, and those over the age of 70 years, pregnant women and people with underlying conditions were asked to self-isolate for at least 7 weeks. However, on 20 March, the four governments of the UK shut all schools, restaurants, pubs, indoor entertainment venues, non-food or non-essential shops, and banned people from meeting anyone they did not live with. People were prohibited from leaving their homes except for one period of exercise per day and to obtain essential food supplies or attend to medical needs. At all times, people were asked to stay at least 2 metres away from those they did not live with (social distancing). On 13 May 2020, some adjustments to the requirements were made in England only, with people being allowed to exercise as much as they wished and to drive to an area to exercise; everyone who could not work from home was urged to return provided they could remain at least 2 metres apart, and plans were made for children to return to school in June. The devolved governments of Wales, Scotland and Northern Ireland maintained their ‘stay at home’ policies. On 14 May 2020, the WHO reported 4 248 389 cases of COVID-19 globally and a worldwide death toll of 292 046.^5^ In the UK, as of 09:00 h on 23 May, over 35 000 people had died having tested positive for the virus,^6,7^ although other sources state that the number of actual deaths of people with symptoms suggesting COVID-19 was much as 55% higher than this number.^8^ Other countries worldwide have had varying numbers of cases and deaths, and widely differing responses to the pandemic.

## Studies of anxiety and depression symptoms since the arrival of SARS-CoV-2

Given that we are facing an unknown and unseen threat to our health and survival, it is unsurprising that there have been increased numbers of people complaining of symptoms of stress, anxiety and depression. A population survey of 1210 respondents from 194 cities in China found that 28.8% of respondents reported moderate to severe symptoms of anxiety, 16.5% reported moderate to severe depressive symptoms and 8.1% reported moderate to severe levels of stress. Almost 85% were spending 20–24 h each day at home, and over 75% were worried about family members contracting COVID-19. Women, students and those who reported poorer general health were more likely to report distress.^9^ Among healthcare workers in China (over 60% from Wuhan), a much higher proportion reported psychological symptoms, with over 70% suffering from distress, more than half having symptoms of depression, and over 44% having symptoms of anxiety. Unsurprisingly, those working on the front line were more likely to report symptoms, as were those working within Hubei Province.^10^ In a Spanish population survey, 18.7% of the sample had depressive symptoms, 21.6% anxiety symptoms and 15.8% post-traumatic stress disorder symptoms. Fewer symptoms were found among older people, those who were economically stable and those who felt they had adequate information about the pandemic. A greater number of symptoms were found in women and those who had symptoms consistent with the virus, and those who had a close relative with symptoms were more likely to report distress. Reported loneliness was also predictive of more psychological symptoms.^11^ A Turkish study using the Hospital Anxiety and Depression Scale^12^ and the Health Anxiety Inventory Health Anxiety^13^ found that almost 24% were above the cut-off to suspect depression, and more than 45% were above the threshold for anxiety. Being a woman, living in an urban area and having a history of psychiatric disorder were found to be risk factors for anxiety, and being female and living in an urban area were risk factors for depression. Women with chronic physical disease and a psychiatric history were at greater risk of health anxiety.^14^

## Discussion

Overall, it can be seen that anxiety and depression are normal emotions existing within the population and experienced to a greater or lesser extent by all people over time. The SARS-CoV-2 pandemic has led to great changes in our way of life, as well as a real fear that we and our loved ones may contract a potentially life-threatening disease. In addition, front-line workers, including healthcare workers, are under increasing stress and heavier workload. It is therefore not surprising that there is an increase in the symptoms of anxiety and depression in the general population, particularly in people working in front-line healthcare. In addition, many people have been indoors with restrictions on physical activity and an inability to visit friends and family. This is even more poignant and damaging as many are not able to be with loved ones at the end of their life and are unable to attend funerals.

The National Health Service has issued guidance for the population to look after their mental health. This includes setting a structure to the day whether or not you are working, making time to speak to friends and family using remote methods, and looking after diet and exercise, as well as restricting the amount of new reporting an individual is watching if this is leading to distress.^15^ Preventive measures such as these may help to reduce some of the symptoms. Indeed, structuring the day and including a balance of activities which give a sense of mastery as well as those that give pleasure can be helpful in combating depressive symptoms.^16^ Ensuring a good balanced healthy diet and adequate hydration, and avoiding smoking, alcohol and drugs are also useful in reducing anxiety and depressive symptoms.^16,17^ Working on sleep hygiene and trying to get a good sleep at night using tried and tested methods can also be useful.^17^ Exercise is also important and known to be beneficial to our mental state^18^. Extreme isolation such as that recommended in the UK for those aged over 70 years and those with severe pre-existing medical conditions may have a detrimental effect not only on physical health and the ability to withstand infection but also on mental health.

## Conclusion

Overall, it can be seen that anxiety and depression are ubiquitous human emotions which occur in response to certain situations and stimuli. These symptoms are usually reversible once the situation changes. However, continued stress at this time may result in longer-term anxiety and depressive syndromes. There are some practical steps we can take to try to limit the effects of the current situation on our own mental health as well as that of our loved ones and our patients.

## About the author

**Lynne M. Drummond**, Consultant Psychiatrist, National Service for OCD and BDD, South West London and St George's Mental Health NHS Trust, London, UK.
